# Is the ADP ribose site of the Chikungunya virus NSP3 Macro domain a target for antiviral approaches?

**DOI:** 10.1016/j.actatropica.2020.105490

**Published:** 2020-04-23

**Authors:** Jacqueline Farinha Shimizu, Daniel Oliveira Silva Martins, Martin J. McPhillie, Grace C. Roberts, Carsten Zothner, Andres Merits, Mark Harris, Ana Carolina Gomes Jardim

**Affiliations:** a São Paulo State University, IBILCE, S. José do Rio Preto, SP, Brazil; b Laboratory of Virology, Institute of Biomedical Science, ICBIM, Federal University of Uberlândia, Uberlândia, MG, Brazil; c School of Chemistry, Faculty of Engineering & Physical Sciences and Astbury Centre for Structural Molecular Biology, University of Leeds, Leeds LS2 9JT, United Kingdom; d School of Molecular and Cellular Biology, Faculty of Biological Sciences and Astbury Centre for Structural Molecular Biology, University of Leeds, Leeds LS2 9JT, United Kingdom; e Institute of Technology, University of Tartu, Nooruse 1, 50411 Tartu, Estonia

**Keywords:** Chikungunya virus, Antiviral, nsP3, Macro domain

## Abstract

Chikungunya virus (CHIKV) is a mosquito-transmitted virus of special concern as it causes Chikungunya fever, characterized by an acute febrile illness, rash, and arthralgia that can progress to chronic and debilitating arthritic symptoms. The effects of climate change on the geographic distribution of the mosquito vector has the potential to expose more of the globe to this virus. No antiviral agents or vaccines are currently available against CHIKV infection and the development of novel therapies that may lead to a future treatment is therefore necessary. In this context, the ADP-ribose binding site of the CHIKV nsP3 macro domain has been reported as a potential target for the development of antivirals. Mutations in the ADP-ribose binding site demonstrated decreased viral replication in cell culture and reduced virulence. In this study, 48,750 small molecules were screened *in silico* for their ability to bind to the ADP-ribose binding site of the CHIKV nsP3 macro domain. From this *in silico* analysis, 12 molecules were selected for *in vitro* analysis using a CHIKV subgenomic replicon in Huh-7 cells. Cell viability and CHIKV replication were evaluated and molecules C5 and C13 demonstrated 53 and 66% inhibition of CHIKV replication, respectively. By using a CHIKV-Dual luciferase replicon contain two reporter genes, we also demonstrated that the treatment with either compounds are probably interfering in the early replication rather than after RNA replication has occurred.

## 1 Introduction

Chikungunya virus (CHIKV) is an arbovirus that belongs to *Alphavirus* genus and *Togaviridae* family (Lumsden, 1955). CHIKV infection can cause fever, skin rash, and arthralgia, and around 15 % of CHIKV infections can be asymptomatic (Miner et al., 2015). The infection can progress to severe symptoms and persist for months or years, leading to an economical burden for many countries (WHO, 2017).

According to the Pan American Health Organization, 349,936 suspected cases were reported in 2016 in the Americas, with 146,914 cases confirmed by laboratory analysis. No commercial antiviral agent or vaccine is available against CHIKV infection. Therefore, the development of an efficient antiviral agent is need to the treatment of infected patients.

CHIKV is an enveloped virus with a positive single strand RNA genome, containing two open reading frames (ORFs). The 5’ ORF encodes four non-structural proteins (nsP1 – nsP4) and the 3’ ORF encodes a subgenomic RNA which encodes the five structural proteins (C, E1, E2, E3 and 6K) (Kumar et al., 2015; Voss et al., 2010).

Of the CHIKV proteins, nsP3 is the most ‘enigmatic’ since its functions in the virus life cycle remain uncertain (Götte et al., 2018). nsP3 comprises 3 domains: an N-terminal region macro domain, followed by the alphavirus unique domain (AUD), and the C-terminal hypervariable region (Malet et al., 2009; Shin et al., 2012). The nsP3 macro domain is conserved among alphaviruses and other pathogenic positive single strand RNA viruses (Eckei et al., 2017). Recently, the ADP-ribosylhydrolase activity of CHIKV nsP3 macro domain was shown to be critical for virus replication and virulence (McPherson et al., 2017).

Previously, the macro domain of CHIKV nsP3 has been described as a potential target in the development of anti-alphavirus compounds (Nguyen et al., 2014; Subudhi et al., 2018; Vijayasri and Hopper, 2017). Despite the interest in the macro domain of nsP3, there is a lack of *in vitro* or *in vivo* studies on drugs that bind to this region of nsP3. Most are computational studies, with no further *in vitro* or in *vivo* validation (Nguyen et al., 2014; Vijayasri and Hopper, 2017). Computational methods can be useful to refine the search for new antiviral drugs, reducing efforts and costs to approve antivirals, however, the validation of these analysis is essential.

In this study, we combined a virtual screening cascade to identify small molecules that putatively bind to ADP-ribose site of the macro domain of nsP3 and further investigated the antiviral activity of the selected molecules *in vitro*.

## 2 Material and Methods

### 2.1 In silico screening

An in-house library containing small synthetic molecules from the Medicinal Chemistry/Chemical Biology (MCCB) group (School of Chemistry, Faculty of Engineering & Physical Sciences - University of Leeds) was docked to the ADP-ribose binding site of the CHIKV nsP3 macro domain (Protein Data Bank - 3GPO) (Malet et al., 2009) using the docking algorithm Glide (vHTS mode). Molecules were ranked according to predicted binding affinity and the top compounds were re-docked using Glide XP mode (Maestro, Schrödinger, LLC, New York, NY, 2017). Then the compounds were visually inspected. Based on these parameters, 12 compounds were selected for *in vitro* activity assays.

### 2.2 Compounds

The 12 compounds selected by *in silico* screening were kindly provided by MCCB group. Compounds were stored at 14°C in 10 mM DMSO stock solutions, in 0% humidity cabinets. After initial *in vitro* assays, new stocks of compounds C5 (ChemDiv V029-0567), C13 (ChemDiv V028-7674) and their respective analogs were purchased (ChemDiv inc, San Diego - US).

### 2.3 Cell lines and replicons

The human hepatoma (Huh-7) and rhabdomyosarcoma (RD) cell lines were grown in DMEM, supplemented with 10% FBS, 0.5 mM non-essential amino acids and 100 units/mL penicillin-streptomycin. Murine myoblast cells (C2C12) were grown in DMEM supplemented with 20% FBS and 100 units/mL penicillin-streptomycin. All cells were maintained at 37°C with 5% CO_2_ in a humidified incubator.

The subgenomic replicons CHIKV wild type nsP3 firefly luciferase (CHIKV-Fluc SGR) and CHIKV dual luciferase (CHIKV-D-Luc SGR) were derived from the ECSA genotype LR2006-OPY1 (Pohjala et al., 2011).

### 2.4 Cell viability assay

Cell viability was measured by MTT [3-(4,5-dimethylthiazol-2-yl)-2,5-diphenyl tetrazolium bromide] (Sigma–Aldrich) assay. 24 hours post treatment, compound-containing media was removed from 96 well plates and MTT at 1 mg/mL solution was added to each well, incubated for 1 hour and replaced with 100 μL of DMSO to solubilize the formazan crystals. The absorbance was measured at 560 nm on Infinite F50 microplate reader (Tecan). Cell viability was calculated according to the equation (T/C) × 100%, which T and C represented the optical density of the treated well and control groups, respectively. DMSO was used as non-treated control. The cytotoxic concentration of 50% (CC_50_) was calculated using Prism (Graph Pad).

### 2.5 RNA Transcription and electroporation

Plasmids were linearized using restriction enzyme NotI-HF (New England Biolabs), prior to *in vitro* transcription using the mMESSAGE mMACHINE™ SP6 Transcription Kit (Invitrogen) following the manufacturer’s protocol. RNA was purified using the LiCl precipitation protocol. For electroporation, 3×10^6^ cells were electroporated with 2 µg of RNA in 4 mm cuvette, 260 V, 25 ms, 1 pulse, using GenePulser Xcell (Bio-Rad) (Roberts et al., 2017). Cells were plated in 48 well plates (6 × 10^4^ cells/well) for replication assays and 96 well plates (2 × 10^4^ cells/well) for cell viability assays. Four hours post-electroporation (h.p.e.) the media was removed, replaced with compound-containing media in different concentrations and incubated for 24 hours.

### 2.6 Replication assay

To measure CHIKV replication levels, compound-containing media was removed 24 hours after treatment, cells were washed with PBS, and harvested by lysis with Passive Lysis Buffer (Promega). Samples were stored at − 20°C and thawed prior to measurement of luminescence levels on a FLUOStar optima microplate reader (BMG Labtech) using the Luciferase Assay System (Promega) for CHIKV-Fluc SGR and the Dual-Luciferase^®^ Reporter system (DLR™) (Promega) for CHIKV-D-Luc SGR. The effective concentration of 50% (EC_50_) was calculated using Prism (Graph Pad). Replication assays were carried out in parallel with cell viability assays.

### 2.7 Statistical Analysis

Individual experiments were performed in triplicate and all assays were performed a minimum of three times in order to confirm the reproducibility of the results. Statistical analysis was performed using GraphPad Prism software. A Kruskal-Wallis test followed by post hoc analysis by Dunn's test was used for multiple comparisons

P values of less than 0.05 (indicated by asterisks) were considered statistically significant.

## 3 Results

### 3.1 In silico identification of ligands to the Macro domain of CHIKV-nsP3

To identify potential inhibitors against the macro domain of CHIKV nsP3, the structure of CHIKV nsP3 complexed with ADP-ribose (Protein Data Bank - 3GPO) was used. The ADP-ribose substrate was manually removed from the ADP-ribose binding site and a library containing 48,750 small synthetic molecules were docked to this region of the protein with Schrodinger's docking tool Glide in vHTS mode using default parameters. The compounds were ranked based on Glide docking scores, a prediction of binding affinity, and the top 1000 scoring compounds were re-docked using Glide XP mode, a higher precision scoring function. After that, the top 50 compounds were visually inspected. And 12 compounds were selected for biological testing based on the docking score (Table 1); inspection of hydrogen bonds between ADP-ribose binding site of CHIKV nsP3 and the compounds; the conformation of each docking pose and the position of the compound inside the binding site relative to the ADP-ribose.

### 3.2 Activity of compounds against CHIKV replication

#### 3.2.1 Initial in vitro activity of compounds selected by docking analysis

**Figure 1:**
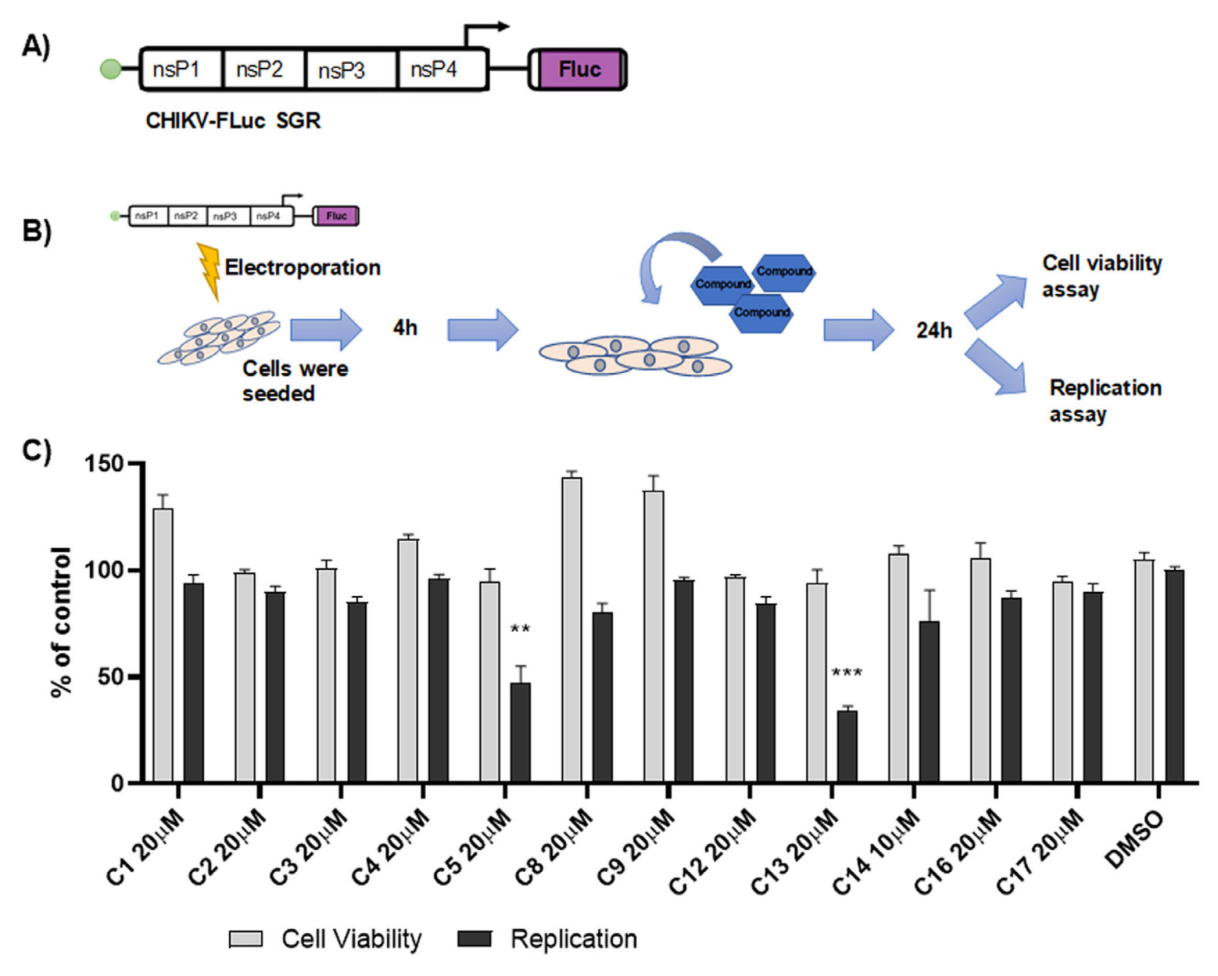
*In vitro* screening of 12 MCCB compounds selected from docking analyses. CHIKV-Fluc SGR replicon scheme **(A)**. Schematic representation of *in vitro* initial trials of MCCB compounds **(B)**. The results of the treatment with maximum non-toxic concentration of Huh-7 cells harbouring CHIKV-Fluc SGR are shown for cell viability and activity against CHIKV replication **(C)**. DMSO was used as non-treated control. The asterisks indicate statistically significant differences between each compound and DMSO control (*p ≤ 0.05, **p ≤ 0.01), calculated using the Kruskal–Wallis test with the Dunn's post test.

The 12 compounds selected by docking analysis were evaluated *in vitro* by their antiviral activity using the CHIKV-Fluc SGR system. Huh-7 cells were electroporated with CHIKV-Fluc SGR RNA, seed in 48 well plates and treated with each compound at 20 μM, 10 μM and 1 μM at 4 h.p.e. to assess the effect of these compounds on both CHIKV replication and cell viability. Figure 1 presents data obtained from the treatment with the maximum non-toxic concentration of each compound. The results demonstrated that at 20 μM compounds C5 and C13 inhibited CHIKV replication by 53% and 66% respectively, and retained 94% of cell viability (Figure 1, Table 1). Other compounds did not demonstrate significant activity against CHIKV replication.

#### 3.2.2 Compounds with anti-CHIKV activity and related analogues

**Figure 2:**
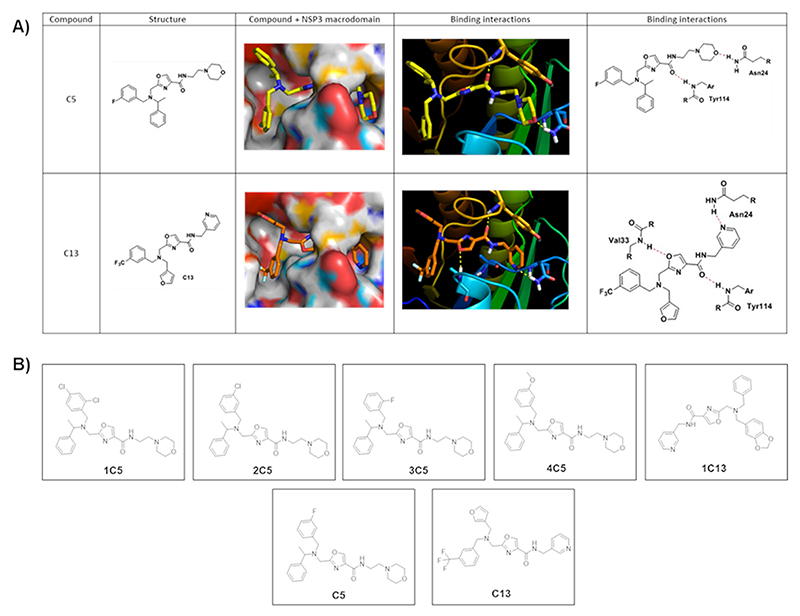
Structure of compounds C5, C13 and analogues. **(A)** Predicted binding conformations of C5 and C13 docked within to the ADP-ribose binding site of CHIKV nsP3 macro domain. Predicted hydrogen bonding interactions between 2D compound structures and CHIKV nsP3 amino acids are shown with a dotted line. **(B)** Molecular structures of the selected analogues (1C5, 2C5, 3C5, 4C5).

*In silico* analysis showed predicted hydrogen bonding interactions of compounds C5 and C13 with residue Tyr114 and van der Waals interactions/hydrophobic effect with residue Val33 and Asn24 (Figure 2A). After initial *in vitro* screening, compounds C5 and C13 were selected for further assays and structural analogues were purchased from ChemDiv (Figure 2B).

**Figure 3:**
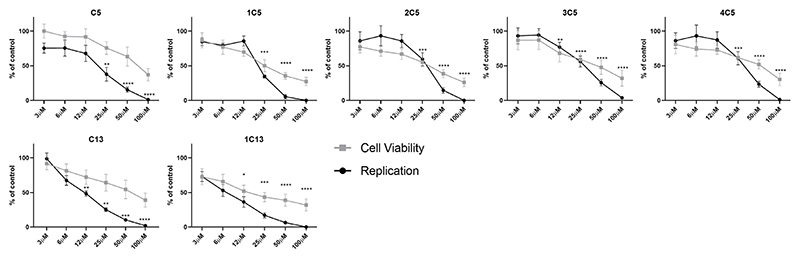
Activity of selected compounds and analogues against CHIKV replication. Huh-7 cells were electroporated with CHIKV-Fluc-SGR RNA, seed and treated 4 h later with compounds at concentrations ranging from 100µM to 3µM. Cell viability (■) was measured by MTT and replication (●) by luciferase assay 24 h post treatment. DMSO was used as non-treated control. P < 0.05 was considered significant, the asterisks indicate statistically significant differences between each compound and DMSO control (*p ≤ 0.05, **p ≤ 0.01, ***p ≤ 0.001 and ****p ≤ 0.0001), calculated using the Kruskal–Wallis test with the Dunn's post test.

The EC_50_ and CC_50_ of C5, C13 and their analogues were determined by performing replication and viability assays in cells treated with a range of concentrations of each compound. Huh-7 cells were electroporated with CHIKV-Fluc SGR RNA, seeded in 48 well plates and treated with a two-fold dilution (ranging from 1.5-100 μM) of each compound at 4 h.p.e. Cell viability and CHIKV replication levels were measured 24 h post treatment (Figure 3). The selective index (SI) was calculated by dividing the value of CC_50_ by EC_50_ (CC_50_/EC_50_). The EC_50_ and CC_50_ values and SI of C5, C13 and their analogues are shown in Table 2. Compound C5 showed EC_50_ of 12.7 μM and CC_50_ of 38.9 μM, and demonstrated lower EC_50_ value than its analogues 1C5, 2C5, 3C5 and 4C5. Compound C13 presented a slightly higher EC_50_ (11.9 μM) and CC_50_ (36.8 μM). While the C13 analogue 1C13 showed a decrease in EC_50_, the value o6 CC_50_ decreased almost by a half.

#### 3.2.3 Investigation of C5 and C13 compounds mechanisms

**Figure 4:**
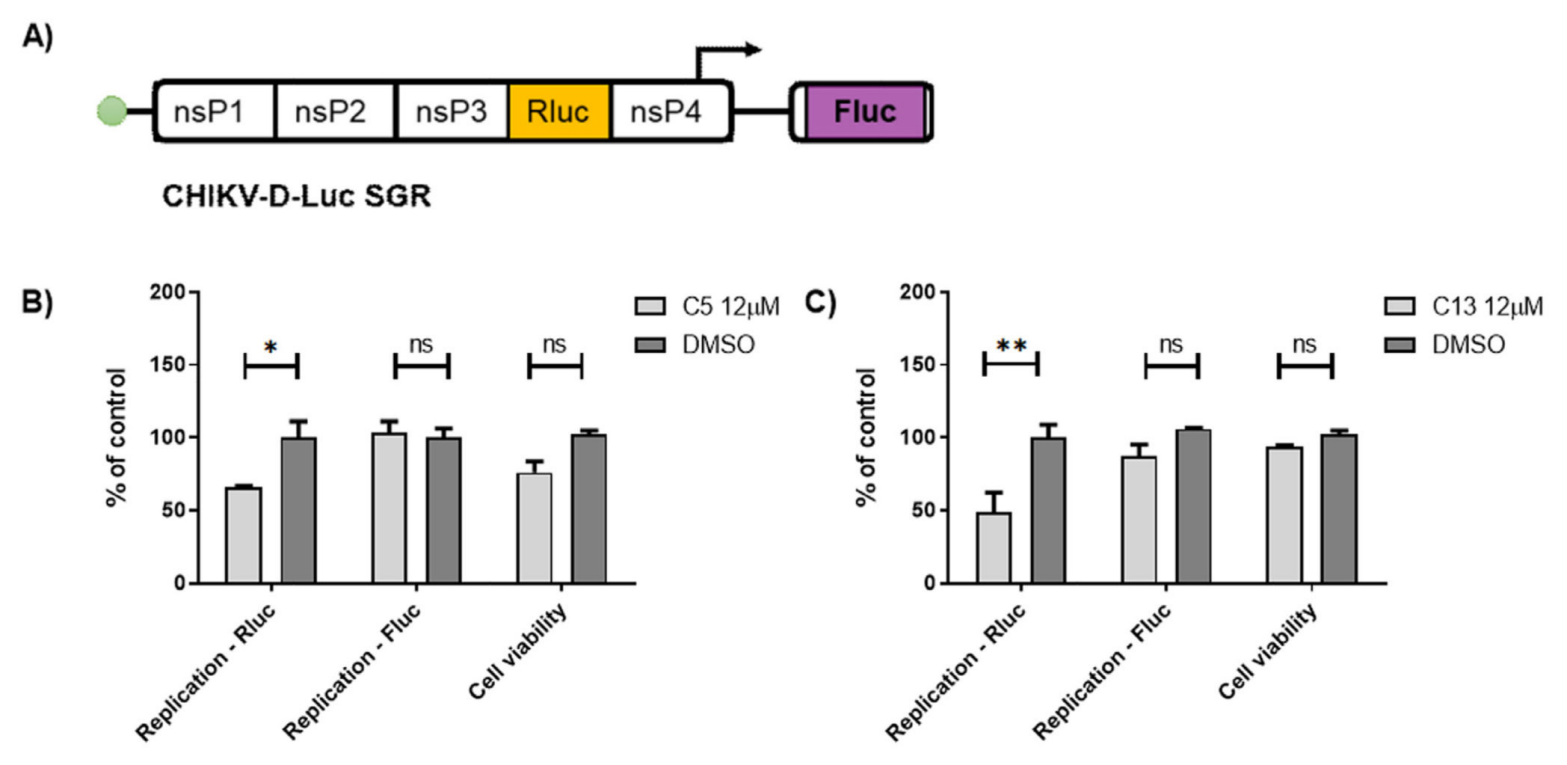
Effect of C13 and C5 at different stages in CHIKV replication. CHIKV-D-Luc wild type RNA **(A)** were electroporated in Huh-7 cells used to analyse the activity of C5 **(B)** and C13 **(C)**. DMSO was used as non-treated control. The asterisks indicate statistically significant differences between each compound and DMSO control (*p ≤ 0.05, **p ≤ 0.01). P < 0.05 was considered significant calculated using the Kruskal–Wallis test with the Dunn’s post test.

Compounds C5 and C13 presented the highest SI and their effects on CHIKV replication were further investigated. To this end, the CHIKV-D-Luc replicon containing two reporter genes, the renilla luciferase (Rluc) and firefly luciferase (Fluc) was used, the replicon is described in (Roberts et al., 2017). The reporter Rluc is present in the hypervariable region of nsP3 of CHIKV and its expression indicate both input translation and early replication levels. Alternatively, the reporter Fluc replaces the virus structural genes and it is only expressed from a subgenomic RNA, thus indicating that RNA replication has occurred (Figure 4A). In order to investigate a possible mechanism of C5 and C13 were acting during early or late replication, Huh-7 cells were electroporated with CHIKV-D-Luc and treated with C5 (Figure 4B) or C13 (Figure 4C) at non-toxic concentrations (concentrations above 80% of cell viability were considerated non-toxic). The data obtained demonstrated that the treatment with C5 (Figure 4B) and C13 (Figure 4C) compound significantly decreased Rluc levels, but not Fluc. Therefore, suggesting that these compounds are most likely interfering with early replication events.

**Figure 5:**
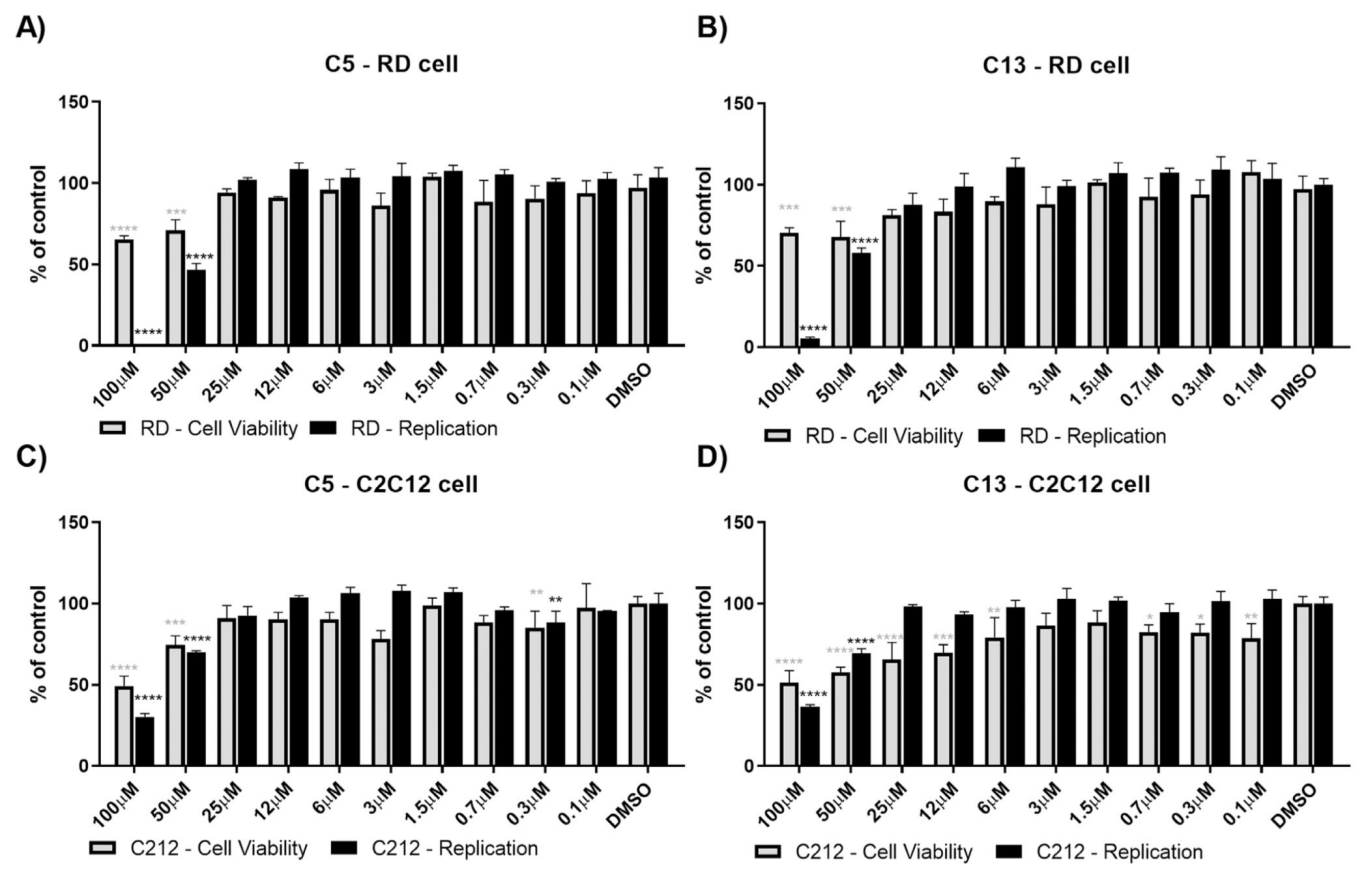
Activity of C5 and C13 in RD and C2C12 cell lines. Cells were electroporated with CHIKV-Fluc RNA, seeded and after 4h treated with C5 or C13. Cell viability and replication were measured in RD (**A** and **B**) and C2C12 cells (**C** and **D**). DMSO was used as non-treated control. The asterisks indicate statistically significant differences between each compound and DMSO control, cell viability differences (grey asterisks) and replication differences (black asterisks). P < 0.05 was considered significant (*p ≤ 0.05, **p ≤ 0.01, ***p ≤ 0.001 and ****p ≤ 0.0001), calculated using the Kruskal–Wallis test with the Dunn's post test.

Finally, the effects of C5 and C13 were also investigated in muscle derived cell lines. For this, human rhabdomyosarcoma (RD) cells and murine myoblast (C2C12) cells were used. However, the results demonstrated that C5 and C13 were only able to inhibit CHIKV replication at concentrations that also demonstrated some cell toxicity (Figure 5). Ats 100 µM, C5 inhibited 100% and 70% of CHIKV replication in RD and C2C12, respectively (cell viability 65% and 49%) (Figure 5A and 5B). C13 inhibited CHIKV replication in 95% and 64% in RD and C2C12, respectively (cell viability 70% and 51%) (Figure 5B and 5D).

## 4 Discussion

Virtual screening has the advantage of reducing costs/time to identify hit compounds when compared to screening large numbers of compounds in an HTS approach (Piccirillo and Amaral, 2018). In this study, the macro domain of CHIKV nsP3 was investigated since this protein has been reviewed and described to participate in several processes of the viral cycle.

The macro domain of CHIKV nsP3 possesses ADP-ribose binding and ADP-ribose hydrolase activity, removing ADP-ribose from mono(ADP-ribosyl)ated proteins, this activity has been shown to be important for viral replication (McPherson et al., 2017). CHIKV mutants with reduced hydrolase activity showed slower virus replication and decrease of virulence, and mutants with no hydrolase activity were unable to replicate in mammalian or mosquito cells (McPherson et al., 2017). Therefore, a compound that can block CHIKV nsP3 hydrolase activity is proposed to be a target for antiviral therapy. Additionally, the macro domain of nsP3 is a well conserved region in the *Alphavirus* genus and other positive single strand RNA viruses, demonstrating the potential to be a broad spectrum antiviral (Eckei et al., 2017; Gregor et al., 2016).

A library of 48,750 small molecules was initially screened *in silico* to predict their ability to bind to ADP-ribose binding site of nsP3. Twelve selected compounds were further investigated *in vitro* for both their toxicity to human hepatoma cells and antiviral activity on CHIKV replication. Compounds C5 and C13 presented the highest selective index (ratio between the cell viability and the replication inhibition) (5.2 and 31, respectively), and demonstrated to share a similar mode of binding to ADP-ribose binding site, in terms of shape and electrostatic potential. Therefore, analogues of C5 and C13 were also investigated, however, they were less active than the original compounds. Moreover, C5 and C13 demonstrated interference in the early steps of CHIKV replication (for example formation of the replication complex) rather than after RNA replication has occurred.

However, most of the computer-aided virtual screening studies based on CHIKV nsP3 describe only *in silico* data (Pérez-Pérez et al., 2019). Nguyen and collaborators used the database Diversity Set II chemical library of the National Cancer Institute (NCI) to virtually screen 1541 compounds in conjunction with the structure of the CHIKV nsP3 macro domain from the Protein Data Bank (PDB id: 3GPG) and selected five potential ligands (Nguyen et al., 2014). Vijayasri and collaborators also used the same CHIKV nsP3 macro domain structure to dock 150 phytochemicals from various plant sources, among them fisetin and quercetin (Vijayasri and Hopper, 2017). Later, the phytochemical fisetin and quercetagetin, compound similar to quercetin, showed *in vitro* anti-CHIKV activity by inhibiting RNA production and viral protein expression (Lani et al., 2016). Baicalein was also considered a potential antiviral candidate against CHIKV nsP3 in a computational study (Seyedi et al., 2016). In 2018, Oo and collaborators showed that baicalein also exhibited binding affinity to CHIKV envelope protein E2. Baicalein present higher inhibition on virus entry than replication. The authors suggested a possible interaction between the compound and cellular factors (Oo et al., 2018).

ADP-ribosylation is a common post-translational modification in cellular proteins (Hottiger et al., 2010). In this regard, the tested compounds showed considerable cytotoxicity, this might be because they were selected to bind to the ADP-ribose binding site of nsP3. By definition this will be similar to the ADP-ribose binding site of cellular macrodomains (Schleicher et al., 2018). ADP-ribosylation is known to regulate cell DNA repair, cell proliferation, transcription and cell death (Barkauskaite et al., 2015; Munnur and Ahel, 2017). Therefore, we suggest that these selected compounds against nsP3 macrodomain may be acting on these other cell pathways affecting cell viability.

In this regard it is interesting to note that compounds C5 and C13 showed higher cytotoxicity in muscle derived cell lines RD and C2C12. Roberts and collaborators, showed that different cell lines present variance in the levels of replication (Roberts et al., 2017). The cytotoxicity observed for RD and C2C12 cell lines could be related to the metabolism of these cells, as well as their ability to support CHIKV replication.

In addition interferon treatment induces the expression of several cellular ADP-ribosyltransferases which act as inhibitors of cellular translation and virus replication (Atasheva et al., 2014), leading to the hypothesis that de-ADP-ribosylation activity of macro domains can act as a response to cellular antiviral mechanisms. Inhibition of nsP3 macro domain activity would therefore have a two-pronged effect – direct inhibition of viral RNA replication and potential blockade of the innate immune response. However, more studies are needed to precisely understand the role(s) of the nsP3 macro domain during the virus life-cycle.

## 5 Conclusion

In this study a virtual screening approach has been used to identify putative CHIKV nsP3 inhibitors that target the ADP-ribose binding site. *In vitro* analysis showed that two of the selected compounds inhibited CHIKV replication, probably acting in the early replication. Computer-aided drug design (CADD) is a facilitator in the process of searching for new antivirals, however, the selection of compounds by docking does not eliminate other variables in a living system and needs to be combined with biological assays.

## Figures and Tables

**Table 1:** Docking scores, physicochemical properties and biological activity of selected ligands docked to ADP-ribose binding site at the macro domain of CHIKV nsP3. HBA = hydrogen bond acceptor. HBD = hydrogen bond donor. MW = molecular weight. PSA = polar surface area.

Compound	Glide Rank	CHIKV %replication 20 uM	% Toxicity (20μM)	Reported Bioactivity	Rotatable bonds	Docking Score	AlogP	logP ZINC15	HBA	HBD	MW	PSA
C9	1	None	None		7	-10,778	1,152		4	2	359,352	91,65
C3	3	None	None		12	-10,371	3,453		5	3	476,548	133,73
C14	8	None	None		12	-9,695	4,193		3	2	484,03	78,51
C1	14	None	None		6	-9,435	1,64		5	1	344,388	114,35
C13	16	66%	None	None	11	-9,388	2,73	4,81	5	1	470,444	84,4
C4	21	None	None		12	-9,153	1,751		6	2	499,531	106,2
C5	30	53%	None	None	11	-8,972	3,025		4	1	466,548	70,84
C17	33	None	None		6	-8,952	1,723		4	2	407,871	117,95
C12	46	None	None		3	-8,751	3,324		2	0	368,857	40,62
C8	48	None	None		9	-8,735	4,065		3	1	413,512	56,15
C16	50	None	None		6	-8,7	1,403		3	2	368,43	81,75

**Table 2:** EC_50_ and CC_50_ of C5 and C13 analogues.

Compound	EC_50_ (μM)	CC_50_ (μM)	SI
C5	12.7	38.9	3.1
1C5	19.8	27.5	1.4
2C5	27.3	27.4	1.0
3C5	26.9	40.5	1.5
4C5	29.3	45.2	1.5
C13	11.6	36.8	3.2
1C13	6.9	18.1	2.6
